# Nicotinamide Mononucleotide Attenuates Inflammatory Activation, Choroidal Neovascularization, and Lesion-Associated Remodeling

**DOI:** 10.1167/iovs.67.10.59

**Published:** 2026-08-25

**Authors:** Jue Wang, Hideto Osada, Steve Chen, Shintaro Yamaguchi, Kaori Hayashi, Kazuno Negishi, Toshihide Kurihara, Norimitsu Ban

**Affiliations:** 1 Laboratory of Aging and Retinal Biology, Keio University School of Medicine, Tokyo, Japan; 2 Department of Ophthalmology, Keio University School of Medicine, Tokyo, Japan; 3 Division of Endocrinology, Metabolism and Nephrology, Keio University School of Medicine, Tokyo, Japan; 4 Laboratory of Photobiology, Keio University School of Medicine, Tokyo, Japan

**Keywords:** choroid neovascularization, NMN, age-related macular degeneration, inflammation

## Abstract

**Purpose:**

To evaluate the therapeutic potential of nicotinamide mononucleotide (NMN) for modulating the neurovascular inflammatory microenvironment and blunting tissue remodeling in neovascular age-related macular degeneration (nAMD).

**Methods:**

A laser-induced choroidal neovascularization (CNV) model was established in C57BL/6J mice, and CNV lesion size was quantified on RPE/choroid flat mounts. Immunostaining evaluated myeloid cell accumulation and fibrosis-associated remodeling. Quantitative RT-PCR and Western blotting assessed inflammatory/angiogenic gene expression and signaling activation in the retina and RPE/choroid. In vitro, lipopolysaccharide (LPS)–stimulated bEnd.3 and primary mouse RPE cells and TGF-β–stimulated THP-1–derived macrophage cells were used to model inflammatory and profibrotic responses.

**Results:**

NMN treatment significantly reduced CNV size in the laser-induced CNV model. This was accompanied by decreased myeloid cell accumulation within CNV lesions. NMN attenuated inflammatory and angiogenesis-related gene expression in the RPE/choroid and neural retina and reduced downstream signaling activation. In vitro, NMN suppressed LPS-induced inflammatory and proangiogenic responses in primary RPE cells and bEnd.3 endothelial cells and inhibited NF-κB activation. NMN further attenuated tissue remodeling, as shown by reduced collagen I–positive area under prolonged and delayed dosing regimens, together with decreased F4/80-positive area and α–smooth muscle actin–positive area within CNV lesions. In TGF-β–induced THP-1–derived macrophage cells, NMN suppressed profibrotic responses.

**Conclusions:**

Our findings indicate that NMN reduces inflammatory signaling and alleviates the inflammatory microenvironment in CNV, accompanied by decreased angiogenesis-related gene expression and fibrosis-related remodeling. By attenuating inflammatory activation and tissue remodeling processes, NMN warrants further evaluation as an adjunctive approach to limit CNV progression and late-stage tissue remodeling in nAMD.

Age-related macular degeneration (AMD) is a leading cause of irreversible vision loss in older adults. The number of individuals with AMD-related vision impairment is projected to reach 21.34 million by 2050, highlighting a growing public health and economic burden.^1^ Neovascular AMD (nAMD), an advanced stage of the disease, is driven by choroidal neovascularization (CNV), in which abnormal vessels invade the subretinal space, causing exudation and hemorrhage, ultimately leading to fibrotic scarring.^2^ Although intravitreal anti-VEGF therapy has become the standard of care, a substantial subset of patients exhibits an incomplete response or develops subretinal fibrosis despite adequate VEGF suppression.^3^^,^^4^ Moreover, inflammation is increasingly recognized as a key contributor to AMD pathogenesis.^5^ Together, these limitations suggest that targeting angiogenesis alone may be insufficient to prevent lesion persistence, chronic inflammation, and scar-forming remodeling, highlighting the need for complementary strategies beyond VEGF suppression.

Accumulating evidence indicates that CNV progression is not simply an angiogenic process but is shaped by a chronic inflammatory microenvironment that promotes both neovascular activity and maladaptive tissue remodeling.^5^^–^^7^ Within this milieu, persistent inflammation can disrupt RPE homeostasis and contribute to photoreceptor degeneration, while also enhancing VEGF production and thereby sustaining pathologic neovascular growth.^8^^,^^9^ Such inflammatory activation is closely coupled to the recruitment and accumulation of immune and glial cells. In particular, infiltrating myeloid cells can amplify inflammatory signaling and maintain a cytokine- and growth factor–rich environment.^10^^,^^11^ Beyond their proangiogenic roles, macrophages can contribute to extracellular matrix deposition and tissue remodeling.^7^ Therefore, modulation of the inflammatory microenvironment and macrophage-associated profibrotic response may represent a strategy to inhibit CNV formation and limit late-phase lesion remodeling.

Nicotinamide mononucleotide (NMN) is a key intermediate in nicotinamide adenine dinucleotide (NAD^+^) biosynthesis. NAD^+^ is a central metabolic cofactor whose levels decline with aging and disease.^12^ NAD^+^ deficiency impairs the metabolic control of inflammatory activation, thereby amplifying downstream inflammatory signaling that promotes excessive cytokine production and chronic pathology.^13^^,^^14^ Pioneering research has demonstrated that long-term NMN administration not only improves ocular function but also attenuates age-linked inflammatory gene expression changes.^13^^,^^15^^–^^17^ In addition, prior studies have reported antifibrotic effects of NMN in nonocular tissues, such as the liver and lung.^18^^,^^19^ However, whether NMN can modulate the complex inflammatory and fibrotic microenvironment associated with nAMD remains to be established.

In this study, we evaluated the therapeutic potential of NMN in a laser-induced CNV mouse model and in lipopolysaccharide (LPS)– or TGF-β–stimulated cell models. We assessed inflammatory and angiogenic signaling, myeloid cell accumulation, and fibrosis-associated lesion remodeling to test the hypothesis that NMN supplementation not only attenuates CNV development but also modulates the inflammatory microenvironment and maladaptive tissue remodeling. Together, these analyses aimed to define the potential of NMN as an adjunctive strategy for nAMD.

## Materials and Methods

### Animals

Six-week-old male C57BL/6J mice (CLEA Japan, Tokyo, Japan) were used in this study. All procedures complied with the ARVO Statement for the Use of Animals in Ophthalmic and Vision Research and were approved by the Keio University Institutional Animal Care and Use Committee (approval number A2021-056). Animals were maintained under controlled temperature (21°C–25°C) and humidity (40%–60%) on a 12-hour light/dark cycle, with food and water provided ad libitum.

### Laser-Induced CNV

Pupils were dilated with 0.5% tropicamide and 0.5% phenylephrine eye drops (Santen Pharmaceutical, Osaka, Japan). General anesthesia was induced by intraperitoneal injection of a mixed anesthetic regimen consisting of midazolam (4 mg/kg body weight [BW]; Sandoz, Tokyo, Japan), medetomidine (0.75 mg/kg BW; Nippon Zenyaku Kogyo, Fukushima, Japan), and butorphanol tartrate (5 mg/kg BW; Meiji Seika Pharma, Tokyo, Japan). Laser photocoagulation was performed using a 532-nm laser system (Lumenis, San Jose, CA, USA) to generate four to five burns (200 mW, 100 ms, 75 µm), placed two disc diameters from the optic nerve head. Only lesions exhibiting a cavitation bubble, indicative of Bruch's membrane rupture, were included for subsequent analyses. The procedure followed our previously reported protocol.^20^

### Treatment With NMN

NMN (Oriental Yeast, Tokyo, Japan) was dissolved in PBS and administered by daily intraperitoneal injection at 500 mg/kg BW according to the experimental timelines. Control mice received vehicle (PBS) at the same injection volume.

### RPE/Choroid Flat-Mount Preparation and Immunofluorescence

Enucleated eyes were fixed in 4% paraformaldehyde for 1 hour at room temperature. Dissected RPE/choroid complexes were postfixed overnight at 4°C, permeabilized with 0.5% Triton X-100, and blocked with 5% BSA. Flat mounts were incubated with FITC-isolectin B4 (IB4) to visualize CNV lesions or with primary antibodies at 4°C overnight followed by secondary antibodies. Nuclei were counterstained with DAPI. Images were acquired using an FV4000 confocal laser scanning microscope (Olympus, Tokyo, Japan). Area and volume were quantified via three-dimensional reconstruction in Imaris (Oxford Instruments, Abingdon, UK). The antibodies used in the study are listed in Table 1.

**Table 1.** Antibodies for Immunostaining and Western Blotting

Antibody	Company	Catalog Number	Dilution
IB4	Vector Laboratories	FL-1201	1:500
Iba1	Wako	019-19471	1:500
CD68	Bio-Rad	MCA1957	1:500
GFAP	Abcam	ab7260	1:500
Collagen I	Abcam	ab316222	1:500
F4/80	Abcam	ab6640	1:500
p-NF-κB	Cell Signaling Technology	3033	1:1000
NF-κB	Cell Signaling Technology	8242	1:1000
p-STAT3	Cell Signaling Technology	9131S	1:1000
STAT3	Santa Cruz Biotechnology	sc-8019	1:200
HSP27	Cell Signaling Technology	2422	1:1000
SIRT1	Sigma-Aldrich	07-131	1:1000
β-Actin	Cell Signaling Technology	4967	1:10,000
Anti–α-SMA	Abcam	ab5694	1:50
Alexa Fluor 488 anti–α-SMA	Abcam	ab184675	1:50
Anti-rabbit Alexa Fluor 488	Thermo Fisher Scientific	A-11008	1:1000
Anti-rabbit Alexa Fluor 555	Thermo Fisher Scientific	A-21434	1:1000
Anti-mouse IgG, HRP-linked antibody	Cell Signaling Technology	7076S	1:10,000
Anti-rabbit IgG, HRP-linked antibody	Cell Signaling Technology	7074S	1:10,000

HRP, horseradish peroxidase.

### Cryosection and Immunofluorescence

Eyes were enucleated, embedded in Optimal Cutting Temperature compound (Sakura Finetek Japan, Tokyo, Japan), and snap-frozen. Serial cryosections (8 µm) were cut using a CryoStar NX70 cryostat (Thermo Fisher Scientific, Waltham, MA, USA). Sections were incubated with primary antibodies and detected with secondary antibodies. Nuclei were counterstained with DAPI. Fluorescence signals were quantified using ImageJ (Rasband, WS, ImageJ; US National Institutes of Health, Bethesda, MD, USA).

### Cell Culture and Treatments of Cell Lines

The mouse brain endothelial cell line (bEnd.3) was cultured in Dulbecco's modified Eagle's medium (DMEM; Nacalai Tesque, Kyoto, Japan); supplemented with 10% fetal bovine serum (Life Technologies, Carlsbad, CA, USA), 100 U/mL penicillin, and 100 µg/mL streptomycin (Fujifilm Wako Pure Chemical, Osaka, Japan); and maintained at 37°C in 5% CO_2_. The human monocytic cell line THP-1 was cultured in RPMI-1640 medium supplemented with 10% fetal bovine serum, 100 U/mL penicillin, 100 µg/mL streptomycin, and 50 µM 2-mercaptoethanol. After 12 hours of serum starvation, bEnd.3 cells were stimulated with LPS (1 µg/mL; Sigma-Aldrich, St. Louis, MO, USA) in the presence or absence of NMN (1 mM). bEnd.3 cells were harvested at 6 hours for quantitative RT-PCR and at 30 minutes for immunofluorescence or Western blotting. THP-1 monocytes were differentiated into macrophages by treatment with 100 ng/mL phorbol 12-myristate 13-acetate (HY-18739; MedChemExpress, Monmouth Junction, NJ, USA) for 24 hours, followed by recovery in growth medium for an additional 24 hours. THP-1–derived macrophages were then stimulated with 10 ng/mL TGF-β1 in the presence or absence of NMN (1 mM) for 96 hours to assess fibrosis-related gene and protein expression.

### Primary Mouse RPE Cell Isolation and Treatment

Primary mouse RPE cells were isolated as previously described.^21^ After incubation in 2% Dispase II at 37°C for 45 minutes, the anterior segment, lens, and iris were removed, and posterior eyecups were incubated in growth medium for 20 minutes. After removal of the neural retina, the posterior eyecups were radially opened, and RPE cells were gently scraped from the underlying choroid using an ophthalmic scraper and collected in growth medium. Growth medium consisted of high-glucose DMEM supplemented with 10% fetal bovine serum, 1% penicillin/streptomycin, 2.5 mM L-glutamine, and 1× MEM nonessential amino acids. For inflammatory stimulation, primary RPE cells were treated with LPS at 50 ng/mL in the presence or absence of NMN and harvested at 24 hours for quantitative RT-PCR analysis or at 2 hours for immunofluorescence staining.

### In Vitro Immunofluorescence

Following LPS stimulation or TGF-β1 stimulation, cells were fixed and permeabilized, then incubated with primary antibodies, followed by secondary antibodies, and nuclei were stained using DAPI. Images were acquired on an FV4000 confocal microscope (Olympus). NF-κB nuclear translocation and α-smooth muscle actin (SMA)– or collagen I–positive areas normalized to DAPI-positive nuclear area were quantified using ImageJ.

### RNA Isolation and Quantitative RT-PCR

Total RNA was extracted using TRI REAGENT (Molecular Research Center, Cincinnati, OH, USA), followed by reverse transcription using the ReverTra Ace qPCR RT kit (Toyobo, Tokyo, Japan). Quantitative RT-PCR was performed using the THUNDERBIRD Next SYBR qPCR Mix (TOYOBO, Osaka, Japan) on a StepOnePlus PCR system (Applied Biosystems, Waltham, MA, USA). Target gene expression was quantified using the 2^−^^ΔΔCt^ method and normalized to GAPDH. The primer sequences are listed in Table 2.

**Table 2.** The Primers for Quantitative RT-PCR

Primer Name	Forward Primer	Reverse Primer
Ms Mmp2	CCTGGACCCTGAAACCGTG	TCCCCATCATGGATTCGAGAA
Ms Hbegf	CGGGGAGTGCAGATACCTG	TTCTCCACTGGTAGAGTCAGC
Ms Il1b	AGGCCACAGGTATTTTGTCG	AGCTCTCCACCTCAATGGAC
Ms Tnfa	GATGAGAGGGAGGCCATTTG	GCCACCACGCTCTTCTGTCTA
Ms Il6	GAGGATACCACTCCCAGACAGACC	AAGTGCATCATCGTTGTCATACA
Ms Icam1	CCTGTTTCCTGCCTCTGAAG	GTCTGCTGAGACCCCTCTTG
Ms Ccl2	TTGGCTCAGCCAGATGCA	CCTACTCATTGGGATCATCTTGC
Ms Vegfa	ACTGGACCCTGGCTTTACTG	TCTGCTCTCCTTCTGTCGTG
Ms Vegfr2	ACGTCGACATAGCCTCCACT	CTCGGTGATGTACACGATGC
Ms Gapdh	GATGACCCTTTTGGCTCCAC	AGGAGCGAGACCCCACTTAAC
Hm α-SMA	CAGAAGGAGATCACGGCCCTA G	CGGCTTCATCGTATTCCTGTT TG
Hm COL1A1	AATGTGGTTCGTGACCGTGA	AGCCTTGGTTGGGGTCAATC
Hm FN1	CGGTGGCTGTCAGTCAAAG	AAACCTCGGCTTCCTCCATAA
Hm MMP2	AGATCTTCTTCTTCAAGGACCGGTT	GGCTGGTCAGTGGCTTGGGGTA
Hm TAGLN	AAGCGCAGGAGCATAAGAGG	ACTGATGATCTGCCGAGGTC
Hm ITGA5	GGTCGGGGGCTTCAACTTA	AGCACACTGACCCCGTCTG
Hm GAPDH	CACCCACTCCTCCACCTTT	TCCACCACCCTGTTGCTGTAG

### Western Blotting

RIPA buffer (Fujifilm Wako Pure Chemical, Osaka, Japan) was used to extract total protein from cells and tissues. Equal amounts of protein were separated by SDS-PAGE and transferred to PVDF membranes. Membranes were blocked and incubated with primary antibodies, followed by horseradish peroxidase–conjugated secondary antibodies. Bands were visualized using a chemiluminescence system and quantified with ImageJ, normalized to β-actin. Raw Western blot images are provided in Supplementary Fig. S2.

### NAD^+^/NADH Ratio Measurement

Retinal NAD^+^/NADH ratios were assessed using an NAD^+^/NADH assay kit (ab65348; Abcam, Cambridge, UK) following the manufacturer's instructions. Six retinas were pooled per sample, homogenized in extraction buffer, and deproteinized using 10-kDa spin columns (ab93349; Abcam). Absorbance at 450 nm was measured using a Cytation 5 plate reader (Agilent Technologies, Santa Clara, CA, USA), and the NAD^+^/NADH ratio was calculated from the derived concentrations.

### Statistical Analysis

All data were analyzed using GraphPad Prism (GraphPad Software, San Diego, CA, USA). Comparisons between two groups were performed using a two-tailed Student's *t*-test, and comparisons among three or more groups were performed using one-way ANOVA followed by Tukey's post hoc test. Data are presented as mean ± SD. *P* values less than 0.05 were considered statistically significant.

## Results

### NMN Attenuates Pathologic Neovascularization in Laser-Induced CNV Mice

**Figure 1.**
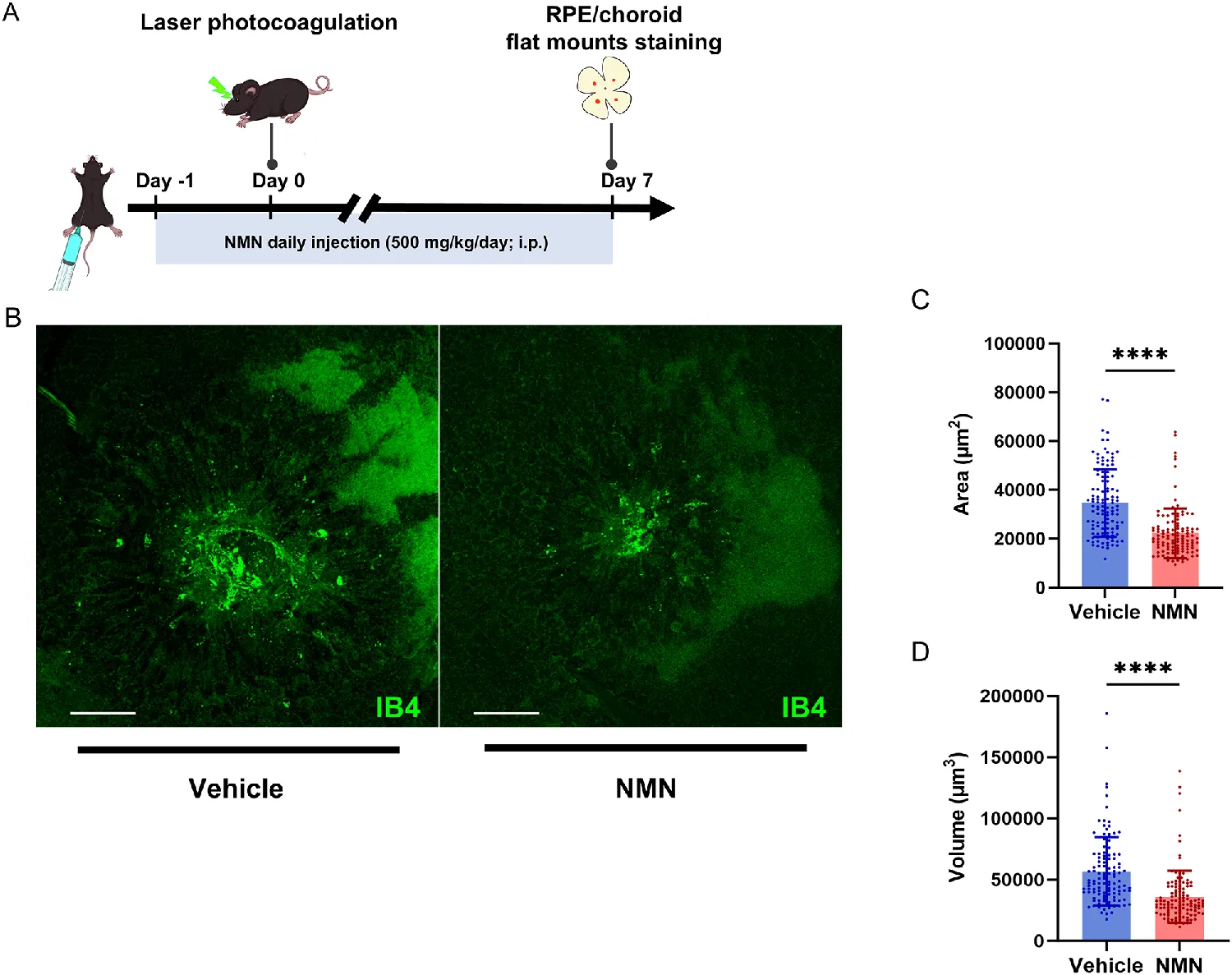
NMN suppresses CNV formation in a laser-induced CNV model. (**A**) Experimental timeline for CNV formation analysis. Laser photocoagulation was performed on day 0. NMN was administered by daily intraperitoneal injection from day –1 to day 7, and eyes were harvested on day 7. (**B**) Representative images of RPE/choroid flat mounts immunostained with isolectin B4 (IB4, *green*). *Scale bar*: 100 µm. (**C**, **D**) Quantification of CNV lesion area and volume (*n* = 116 and 113, respectively). Data are presented as mean ± SD. Student's *t*-test in **C** and **D**. ****P < 0.0001.

To evaluate the therapeutic potential of NMN against pathologic angiogenesis, we employed a laser-induced CNV mouse model. Following a continuous treatment protocol initiated 1 day prior to laser photocoagulation (Fig. 1A), eyes were enucleated on postlaser day 7. RPE/choroid flat mounts were prepared and stained with IB4 to visualize neovascular structures (Fig. 1B). Quantitative analysis revealed that NMN treatment significantly reduced both CNV lesion area and volume compared with the vehicle group (Figs. 1C, 1D). These findings suggest that NMN administration reduces CNV development.

### NMN Attenuates Myeloid Cell Accumulation in Laser-Induced CNV Mice

**Figure 2.**
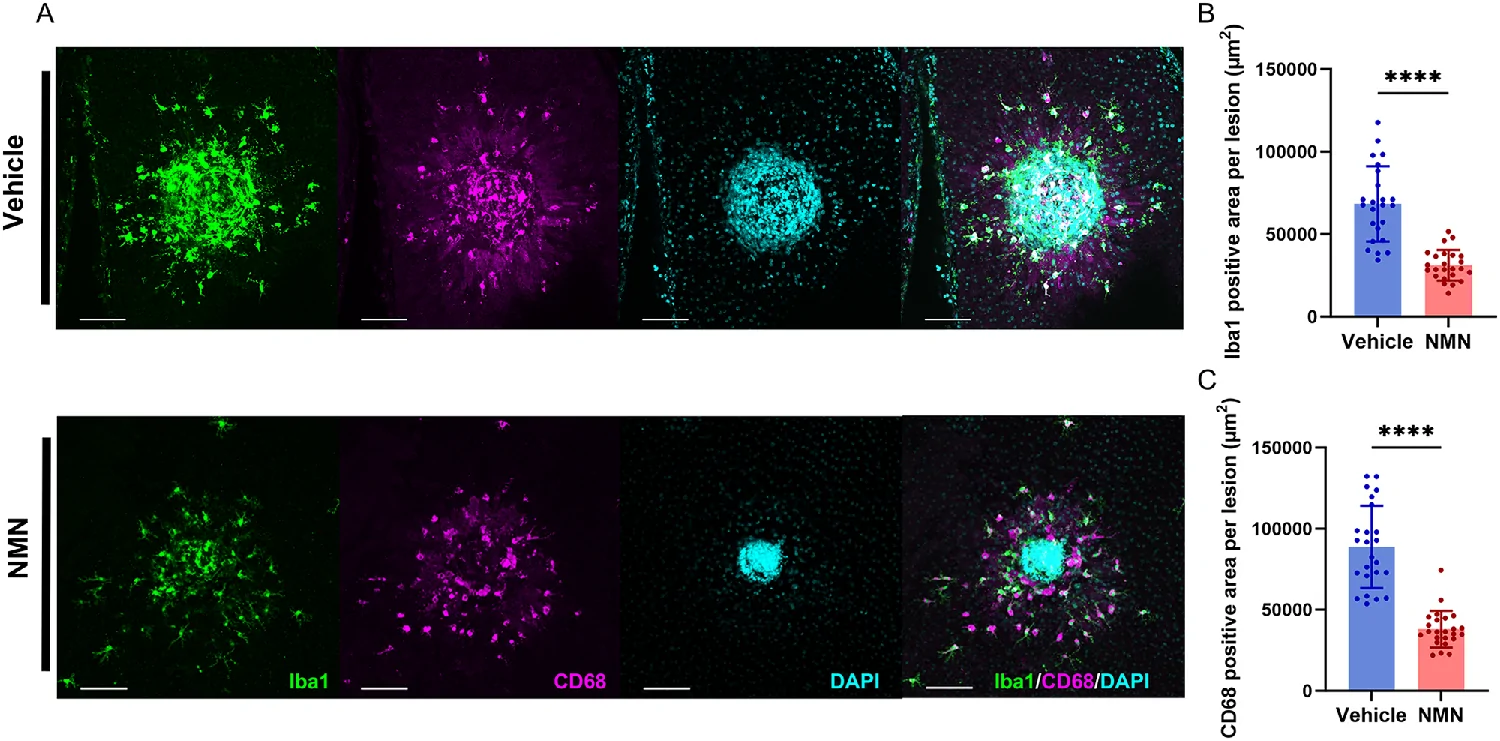
NMN mitigates myeloid cell accumulation in a laser-induced CNV model. (**A**) Representative images of RPE/choroid flat mounts immunostained with Iba1 (*green*), CD68 (*magenta*), and DAPI (*cyan*). *Scale bar*: 100 µm. (**B**, **C**) Quantification of Iba1-positive (**B**) and CD68-positive (**C**) area per lesion (*n* = 24 and 24, respectively). Data are presented as mean ± SD. Student's *t*-test in **B** and **C**. ****P < 0.0001.

Based on the evidence that myeloid cell accumulation exacerbates CNV pathology,^10^ we investigated whether NMN attenuated these responses. Immunostaining of RPE/choroid flat mounts for the microglial marker Iba1 and the macrophage marker CD68 (Fig. 2A) revealed that NMN treatment significantly reduced the Iba1- and CD68-positive area (Figs. 2B, 2C), indicating decreased microglia/macrophage accumulation. These findings demonstrate that NMN treatment is associated with reduced immune cell accumulation within the CNV microenvironment.

### NMN Attenuates Inflammatory and Angiogenic Signaling in Laser-Induced CNV Mice

**Figure 3.**
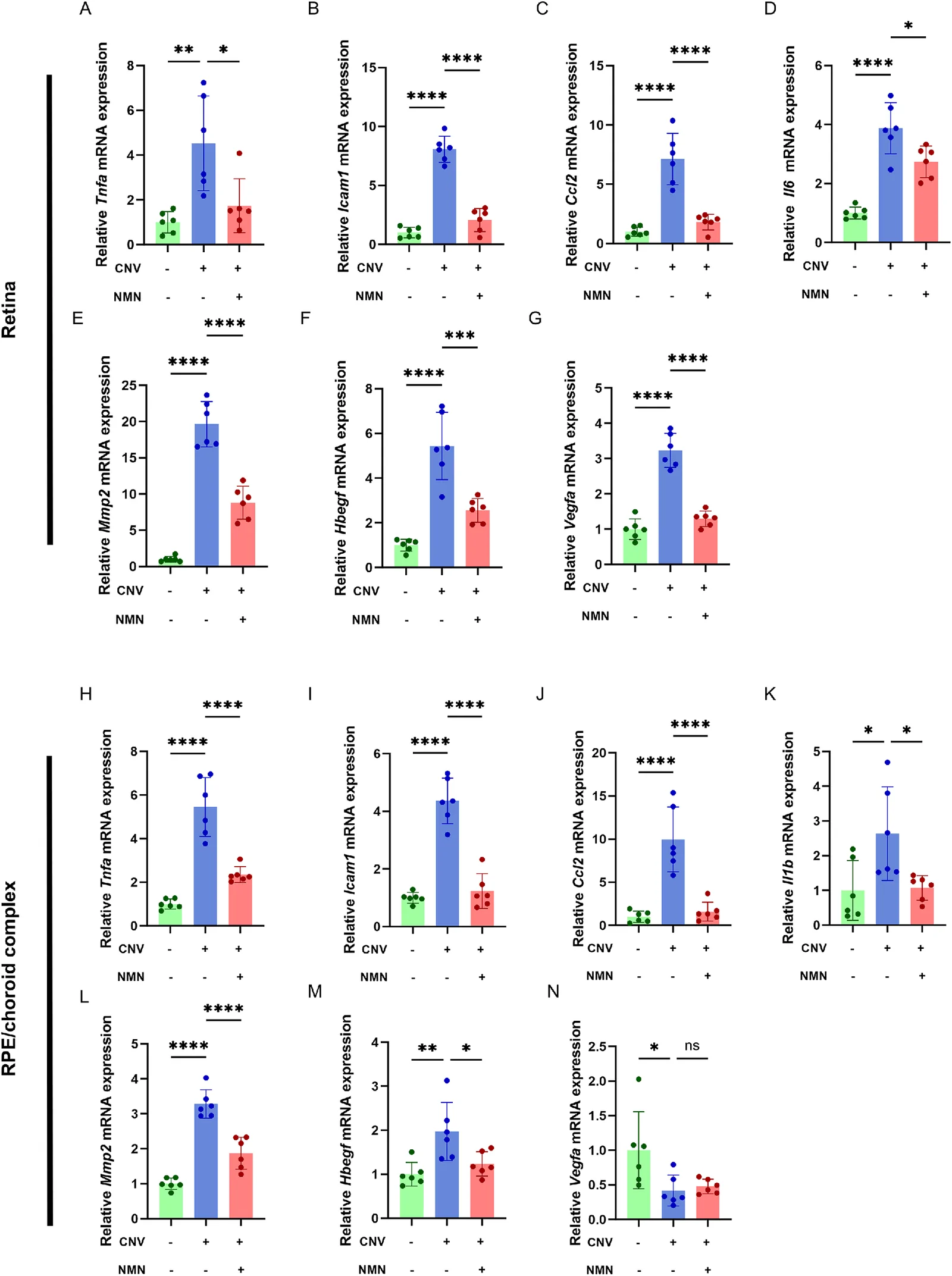
NMN attenuates inflammatory and angiogenic signaling in the neural retina and RPE/choroid in a laser-induced CNV model. (**A–N**) Relative mRNA expression of inflammatory and angiogenic genes in the neural retina (*Tnfa*, *Icam1*, *Ccl2*, *Il6*, *Mmp2*, *Hbegf*, *Vegfa*; **A–G**) and RPE/choroid (*Tnfa*, *Icam1*, *Ccl2*, *Il1b*, *Mmp2*, *Hbegf*, *Vegfa*; **H–N**) (*n* = 6). Data are presented as mean ± SD. One-way ANOVA in **A–N**. *P < 0.05, **P < 0.01, ***P < 0.001, ****P < 0.0001; ns, not significant.

**Figure 4.**
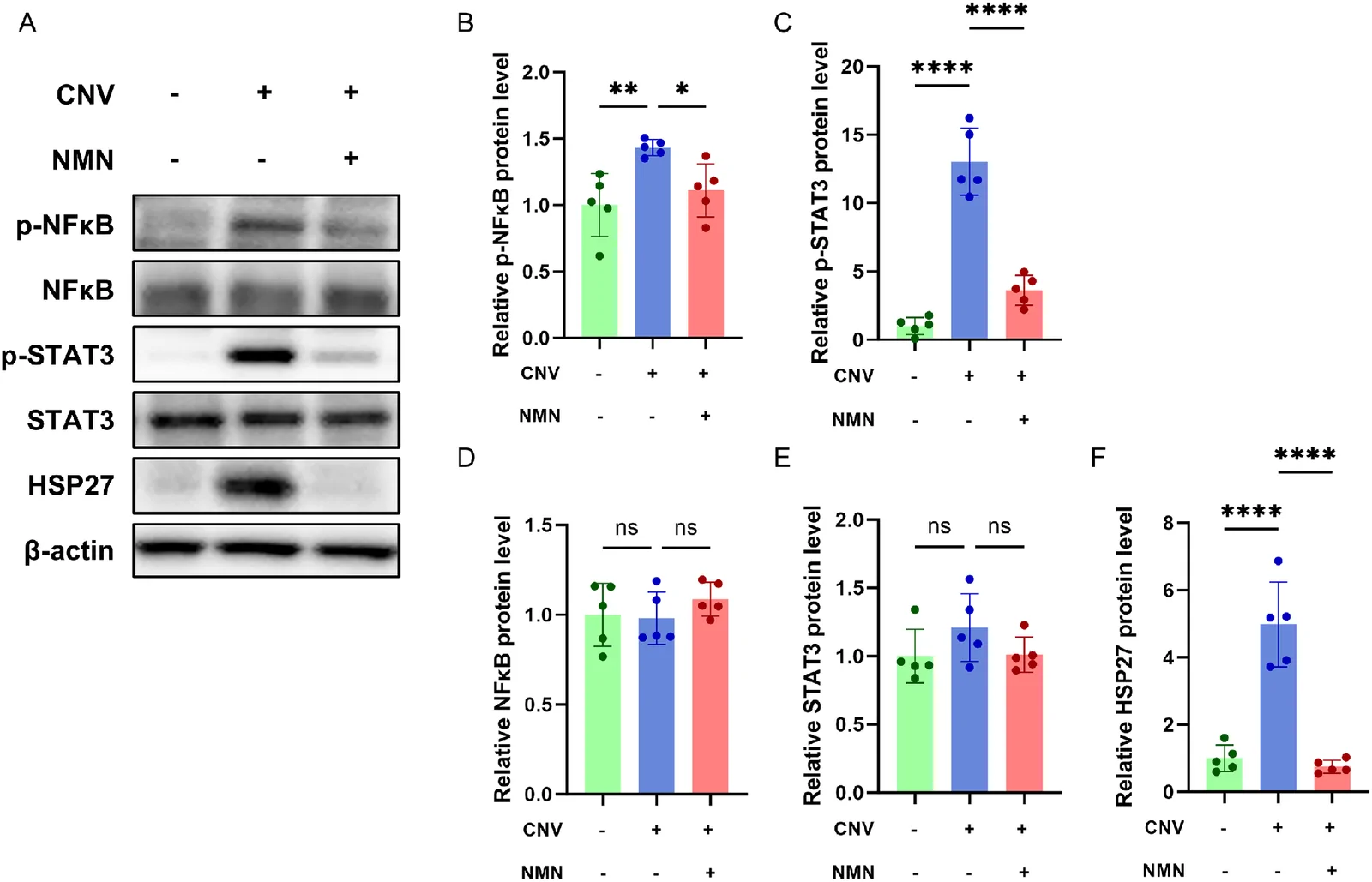
NMN suppresses inflammatory signaling in the neural retina in a laser-induced CNV mouse model. (**A–F**) Western blot analysis of the neural retina. Representative immunoblots are shown in **A**. Densitometric quantification of p-NF-κB, p-STAT3, total NF-κB, total STAT3, and HSP27 is shown in **B–F** (*n* = 5). Proteins were normalized to β-actin. Data are presented as mean ± SD. One-way ANOVA in **B–F**. *P < 0.05, **P < 0.01, ****P < 0.0001; ns, not significant.

Given that NAD^+^-dependent metabolic and inflammatory signaling has been implicated in the regulation of pathologic angiogenesis,^22^ we next examined whether NMN treatment modulated retinal NAD^+^ metabolism in vivo. NMN administration significantly increased retinal NAD^+^ levels, accompanied by an increase in SIRT1 protein expression (Supplementary Fig. S1). We then assessed angiogenic and inflammatory responses in ocular tissues. In the neural retina, NMN reduced mRNA expression of inflammatory cytokines (*Tnfa*, *Icam1*, *Ccl2*, *Il6*; Figs. 3A–D) and angiogenesis-related factors (*Mmp2*, *Hbegf*, *Vegfa*; Figs. 3E–G). In the RPE/choroid complex, NMN decreased inflammatory mediators (*Tnfa*, *Icam1*, *Ccl2*, *Il1b*; Figs. 3H–K) and angiogenesis-related factors (*Mmp2* and *Hbegf*; Figs. 3L, 3M). In contrast, *Vegfa* expression was decreased after CNV induction (Fig. 3N). At the protein level, Western blot analysis of neural retina showed that NMN attenuated phosphorylation of NF-κB and STAT3 (Figs. 4A–C) without affecting total NF-κB or STAT3 levels (Figs. 4A, 4D, 4E). HSP27 protein levels were also reduced (Figs. 4A, 4F). Collectively, these findings suggest that NMN attenuates CNV-associated inflammatory and angiogenic signaling in vivo.

### NMN Suppresses LPS-Induced Inflammatory and Angiogenic Responses In Vitro

**Figure 5.**
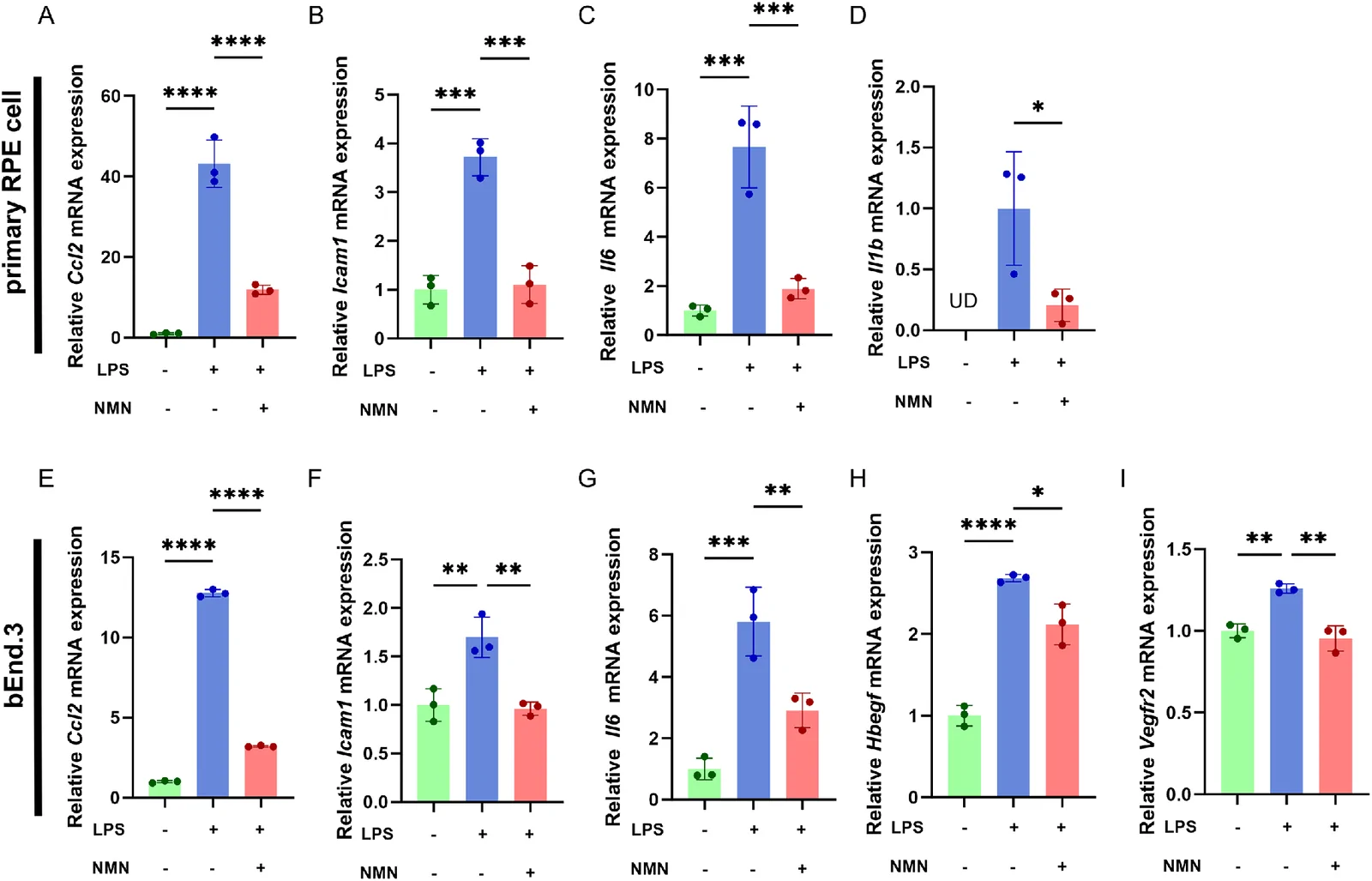
NMN suppresses LPS-induced mRNA expression of inflammatory and angiogenic genes in vitro. (**A**–**D**) Relative mRNA expression of inflammatory genes in primary mouse RPE cells (*Ccl2*, *Icam1*, *Il6*, *Il1b*) (*n* = 3). (**E–I**) Relative mRNA expression of inflammatory and angiogenic genes in bEnd.3 cells (*Ccl2*, *Icam1*, *Il6*, *Hbegf*, *Vegfr2*) (*n* = 3). Data are presented as mean ± SD. One-way ANOVA in **A–C** and **E–I**; Student's *t*-test in **D**. *P < 0.05, **P < 0.01, ***P < 0.001, ****P < 0.0001. UD, undetermined.

We further validated the anti-inflammatory and antiangiogenic properties of NMN using an LPS-induced in vitro inflammation model. Primary mouse RPE cells were included as a physiologically relevant RPE model to evaluate inflammatory responses, and bEnd.3 cells were used to assess vascular endothelial activation. In primary RPE cells, NMN mitigated the LPS-induced expression of inflammatory mediators, including *Ccl2*, *Icam1*, *Il6*, and *Il1b* (Figs. 5A–D). In bEnd.3 cells, LPS stimulation induced a marked upregulation of genes associated with inflammation and angiogenesis, including *Ccl2*, *Icam1*, *Il6*, *Hbegf*, and *Vegfr2*. NMN treatment significantly attenuated this upregulation (Figs. 5E–I). Collectively, these data provide in vitro evidence supporting the ability of NMN to downregulate pathologic gene expression linked to inflammation and angiogenesis.

### NMN Suppresses LPS-Induced NF-κB Activation In Vitro

**Figure 6.**
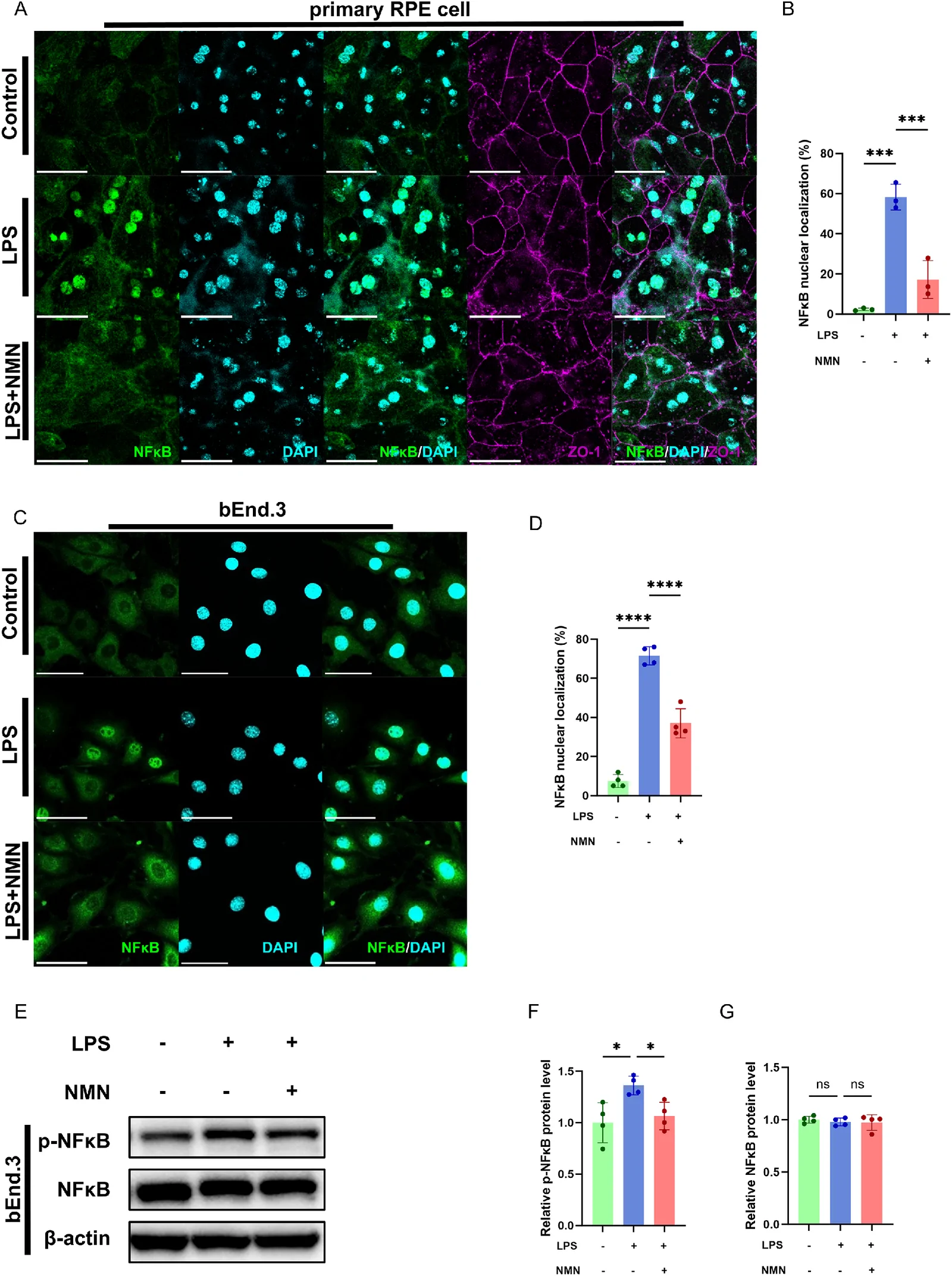
NMN inhibits LPS-induced NF-κB activation in vitro. (**A**) Representative images of primary RPE cells immunostained with NF-κB (*green*), ZO-1 (*magenta*), and DAPI (*cyan*). *Scale bar*: 50 µm. (**B**) Quantification of NF-κB nuclear localization (*n* = 3). (**C**) Representative images of bEnd.3 cells immunostained with NF-κB (*green*) and DAPI (*cyan*). *Scale bar*: 50 µm. (**D**) Quantification of NF-κB nuclear localization (*n* = 4). (**E–G**) Western blot analysis of bEnd.3 cells. Representative immunoblots are shown in **E**. Densitometric quantification of p-NF-κB and total NF-κB is shown in **F** and **G** (*n* = 4). Proteins were normalized to β-actin. Data are presented as mean ± SD. One-way ANOVA in **B**, **D**, **F**, and **G**. *P < 0.05, ***P < 0.001, ****P < 0.0001; ns, not significant.

NF-κB signaling contributes to inflammatory and angiogenic processes in AMD.^23^ We therefore examined whether NMN modulated NF-κB activation in LPS-stimulated primary RPE cells and bEnd.3 cells. In primary RPE cells, ZO-1 immunostaining confirmed the characteristic epithelial monolayer morphology of the cultured cells, and immunofluorescence analysis showed that NMN reduced LPS-induced nuclear translocation of NF-κB (Figs. 6A, 6B). A similar effect was observed in bEnd.3 cells, where NMN reduced LPS-induced nuclear translocation of NF-κB (Figs. 6C, 6D). Western blot analysis in bEnd.3 cells demonstrated that NMN suppressed LPS-induced NF-κB phosphorylation while total NF-κB levels remained unchanged (Figs. 6E–G). These data indicate that NMN inhibits LPS-induced NF-κB signaling in vitro.

### NMN Attenuates Collagen I Deposition in Laser-Induced CNV Mice

**Figure 7.**
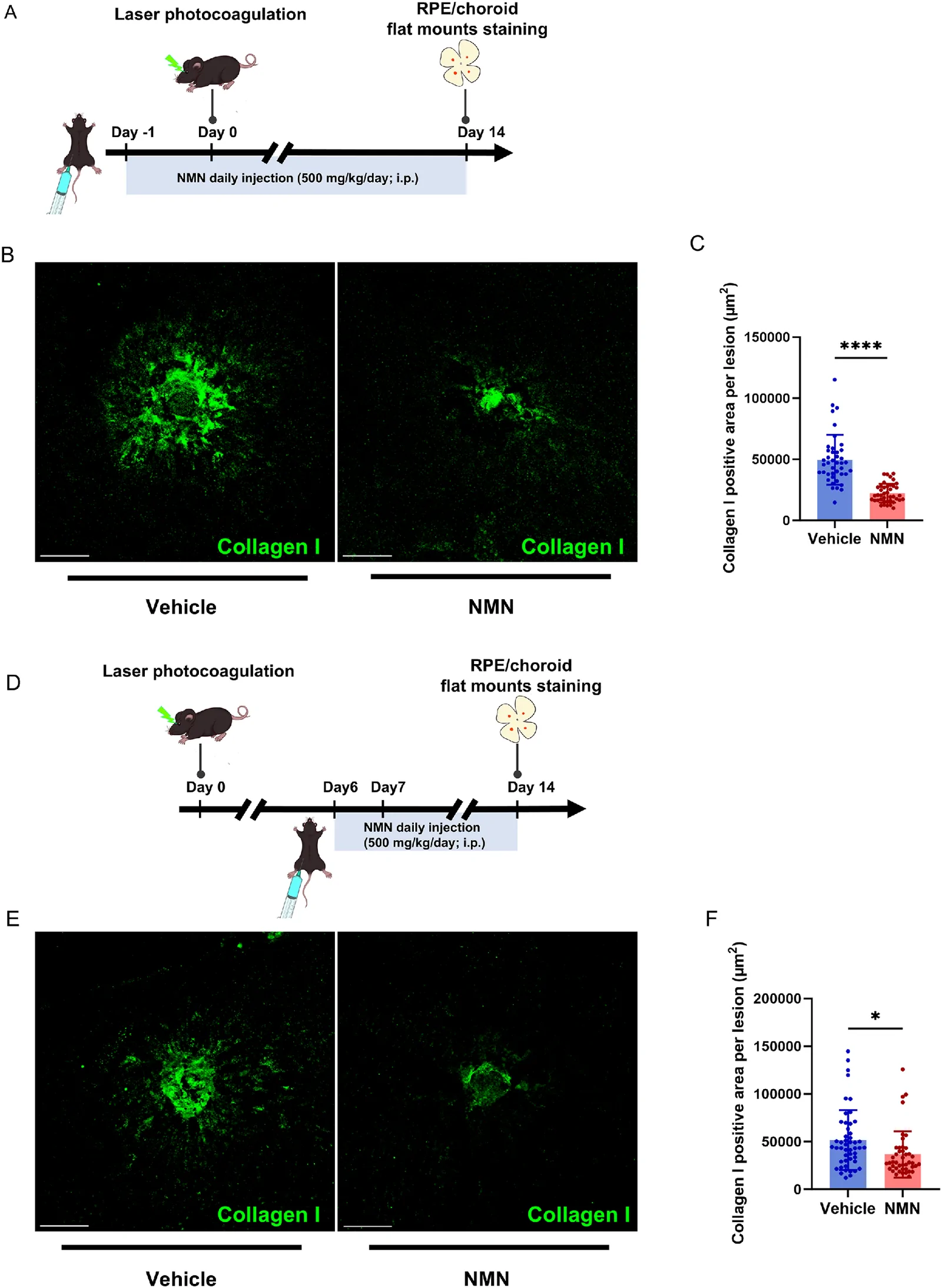
NMN administration suppresses collagen I deposition in a laser-induced CNV model. (**A–C**) Prolonged NMN treatment. (**A**) Experimental timeline. Laser photocoagulation was performed on day 0. NMN was administered by daily intraperitoneal injection from day –1 to day 14, and eyes were harvested on day 14. (**B**) Representative images of RPE/choroid flat mounts immunostained with collagen I (*green*). *Scale bar*: 100 µm. (**C**) Quantification of collagen I–positive area (*n* = 42 and 41, respectively). (**D–F**) Delayed NMN treatment. (**D**) Experimental timeline. For delayed treatment, NMN was administered by daily intraperitoneal injection from day 6 to day 14, and eyes were harvested on day 14. (**E**) Representative images of RPE/choroid flat mounts immunostained with collagen I (*green*). *Scale bar*: 100 µm. (**F**) Quantification of collagen I–positive area (*n* = 49 and 44, respectively). Data are presented as mean ± SD. Student's *t*-test in **C** and **F**. *P < 0.05, ****P < 0.0001.

Subretinal fibrosis represents a late adverse outcome associated with irreversible vision loss in nAMD.^3^ Given that mouse laser-induced CNV lesions can spontaneously undergo remodeling, we assessed collagen I deposition as a marker of lesion-associated extracellular matrix remodeling. Under a prolonged treatment protocol, NMN was administered daily from 1 day before photocoagulation through postlaser day 14 (Fig. 7A). Collagen I immunostaining of RPE/choroid flat mounts showed that NMN reduced the collagen I–positive area compared with the vehicle group (Figs. 7B, 7C), suggesting reduced collagen I–associated remodeling during lesion resolution. Because CNV activity generally peaks around day 7 and precedes a later fibrotic remodeling phase in the mouse laser CNV model,^24^ we employed a delayed treatment protocol to minimize the influence of NMN on initial CNV lesion size and thereby assess the impact of NMN on subsequent lesion-associated remodeling. NMN treatment was initiated on day 6 and continued through day 14 (Fig. 7D). Notably, delayed NMN administration still significantly reduced collagen I–positive area per lesion (Figs. 7E, 7F). Together, these findings suggest that NMN attenuates lesion-associated collagen I deposition in laser-induced CNV. The efficacy observed with delayed treatment further supports an effect during the later remodeling phase.

### NMN Attenuates Macrophage Accumulation and Tissue Remodeling in Laser-Induced CNV Mice

**Figure 8.**
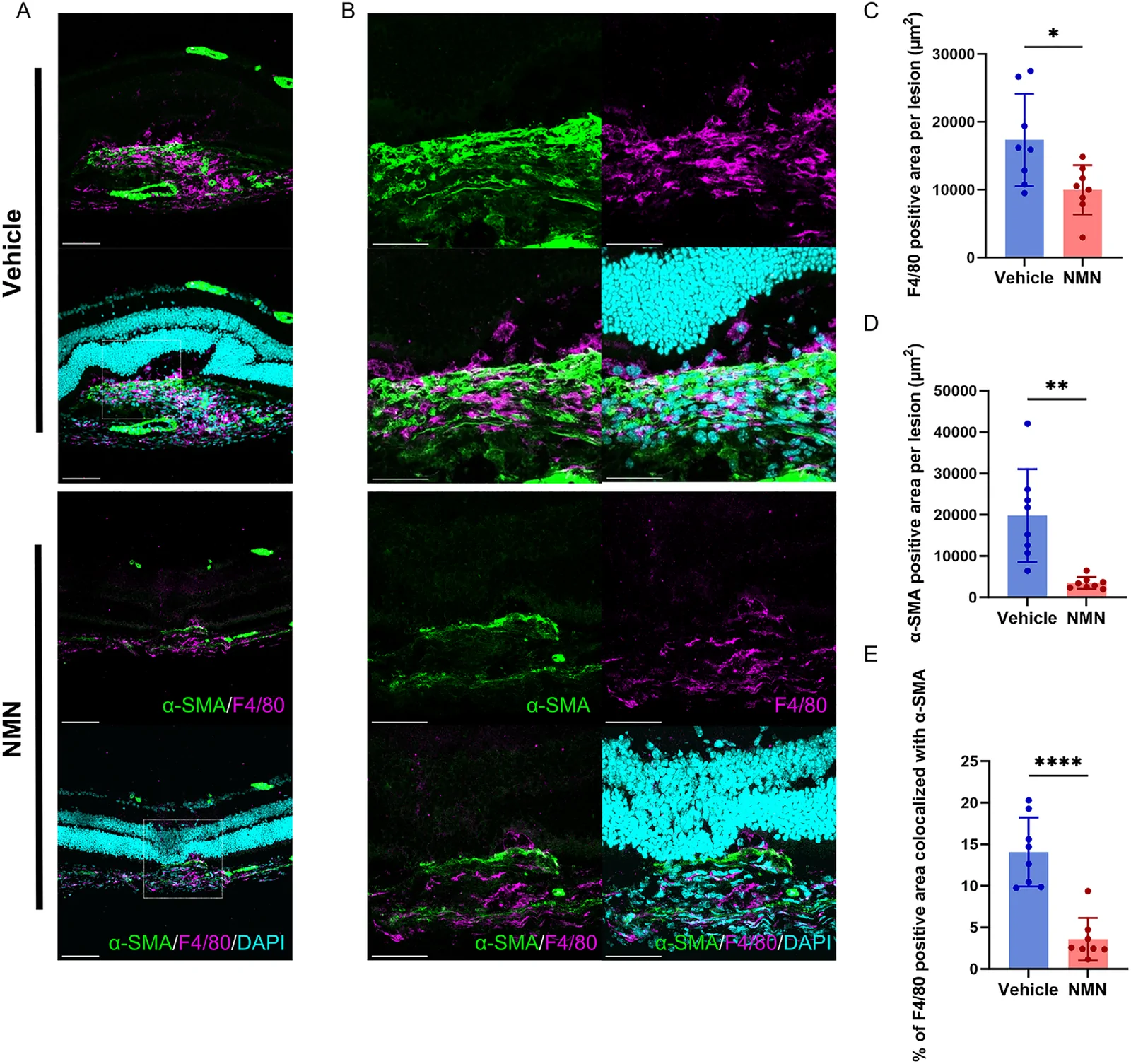
NMN reduces macrophage accumulation and α-SMA–associated remodeling in a laser-induced CNV model. (**A**) Representative images of retinal cryosection immunostained for α-SMA (*green*), F4/80 (*magenta*), and DAPI (*cyan*). *Scale bar*: 100 µm. (**B**) Magnified views of the boxed regions in **A**. *Scale bar*: 50 µm. (**C**) Quantification of F4/80-positive area per lesion (*n* = 8 and 8, respectively). (**D**) Quantification of α-SMA–positive area per lesion (*n* = 8 and 8, respectively). (**E**) Quantification of colocalization, calculated as the percentage of F4/80-positive area colocalized with α-SMA (*n* = 8 and 8, respectively). Data are presented as mean ± SD. Student's *t*-test in **C–E**. *P < 0.05, **P < 0.01, ****P < 0.0001.

To evaluate whether NMN affects fibrosis-associated remodeling in CNV lesions, retinal sections were immunostained for F4/80 and α-SMA. In vehicle-treated CNV eyes, F4/80-positive macrophage accumulation and α-SMA–positive remodeling signals were clearly observed within the lesion area (Figs. 8A, 8B). NMN treatment significantly reduced both the F4/80-positive area and the α-SMA–positive area per lesion (Figs. 8C, 8D). The overlap between F4/80-positive and α-SMA–positive signals was also reduced after NMN treatment (Fig. 8E). These results indicate that NMN treatment is associated with reduced macrophage accumulation and tissue remodeling in CNV lesions.

### NMN Suppresses TGF-β–Induced Fibrotic Gene Expression In Vitro

**Figure 9.**
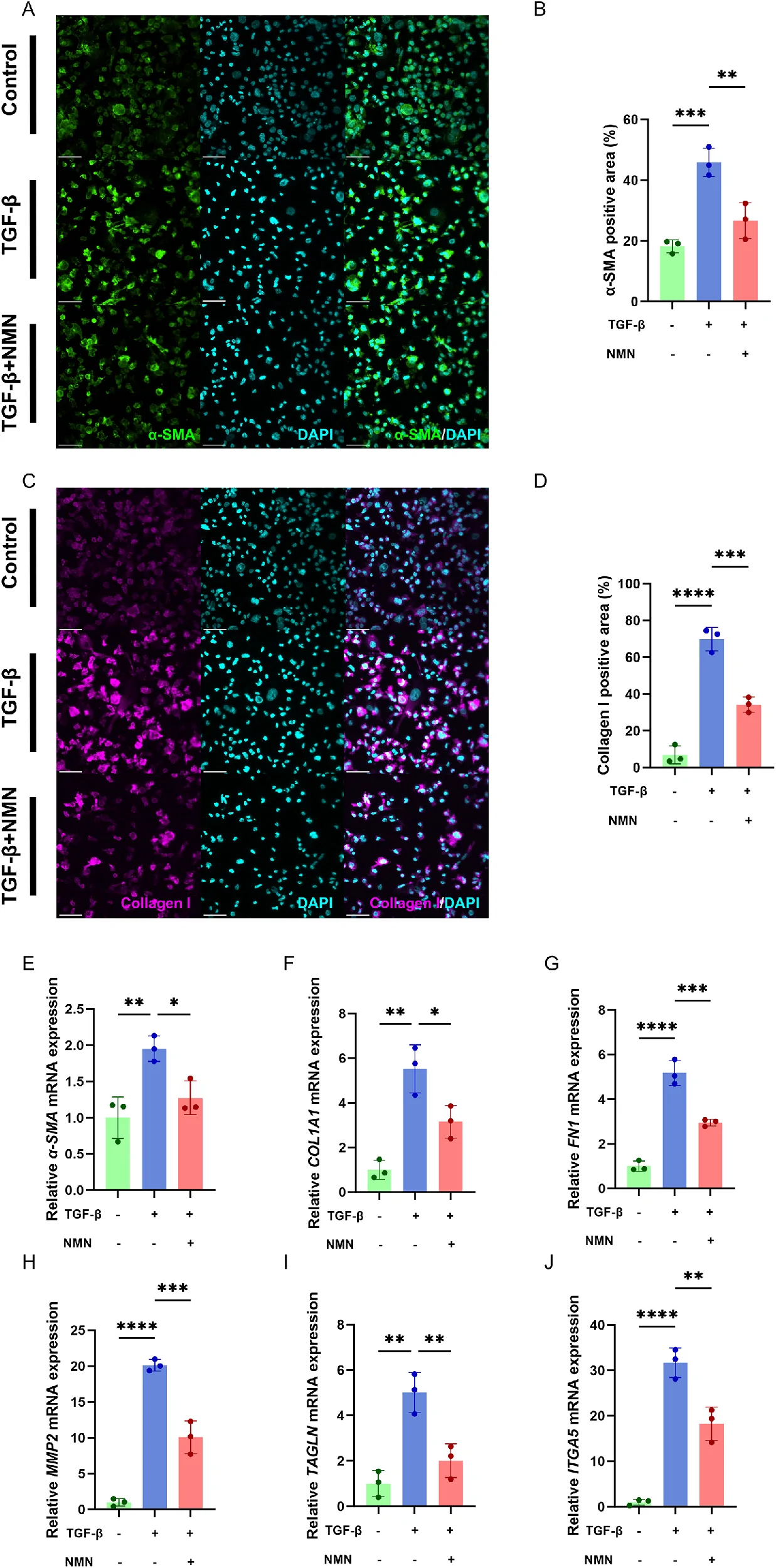
NMN suppresses TGF-β–induced fibrosis-related marker expression in vitro. (**A**, **C**) Representative images of THP-1–derived macrophage cells immunostained with α-SMA (*green*), collagen I (*magenta*), and DAPI (*cyan*). *Scale bar*: 50 µm. (**B**, **D**) Quantification of α-SMA–positive area (**B**) and collagen I–positive area (**D**) normalized to DAPI-positive nuclear area (*n* = 3). (**E–J**) Relative mRNA expression of *α-SMA*, *COL1A1*, *FN1*, *MMP2*, *TAGLN*, and *ITGA5* in THP-1 cells treated with TGF-β and NMN (*n* = 3). Data are presented as mean ± SD. One-way ANOVA in **B**, **D**, and **E–J**. *P < 0.05, **P < 0.01, ***P < 0.001, ****P < 0.0001.

Given that TGF-β is a key mediator of profibrotic macrophage activation and tissue fibrosis,^25^ we used TGF-β–stimulated THP-1–derived macrophages to determine whether NMN modulates the profibrotic response. Immunocytochemistry showed increased α-SMA and collagen I expression after TGF-β stimulation, and these increases were reduced by NMN treatment (Figs. 9A–D). Consistently, TGF-β stimulation induced upregulation of fibrosis-related genes (*α-SMA*, *COL1A1*, *FN1*, *MMP2*, *TAGLN*, and *ITGA5;* Figs. 9E–J), which was attenuated by NMN treatment. These results suggest that NMN suppresses TGF-β–induced fibrotic response in THP-1 cells.

## Discussion

Although nAMD is often characterized by choroidal neovascularization, it is also accompanied by a chronic inflammatory microenvironment that can promote lesion persistence, tissue remodeling, and subretinal fibrosis.^5^^–^^7^ While anti-VEGF therapy effectively suppresses angiogenic activity, long-term visual outcomes remain suboptimal in a substantial subset of patients.^4^ Persistent inflammation and maladaptive tissue remodeling contribute to ongoing disease activity and may drive resistance to anti-VEGF therapy.^3^^,^^26^^,^^27^ These limitations underscore the need for therapeutic strategies that complement anti-VEGF therapy by modulating disease programs beyond VEGF-driven angiogenesis.

In the present study, we demonstrated that NMN significantly reduced CNV lesion area and volume in a mouse laser-induced CNV model. This was accompanied by decreased *Vegfa* expression in the neural retina. Although *Vegfa* expression in the RPE/choroid complex was not significantly altered by NMN treatment, *Vegfa* mRNA levels in this tissue may not reflect local VEGF-A protein accumulation or VEGF-dependent angiogenic activity within CNV lesions.^28^ We next used an LPS-stimulated in vitro inflammation model and found that NMN reduced inflammatory activation and downregulated angiogenesis-related genes, including *Vegfr2* and *Hbegf*, in bEnd.3 cells. Together, these results suggest that NMN modulates VEGF-related angiogenic programs at multiple regulatory levels, rather than acting on a single node.^29^ Notably, prior studies have suggested that NAD^+^ could promote angiogenesis in other tissues.^30^^,^^31^ However, such proangiogenic phenotypes are reported mainly in contexts such as ischemic repair or homeostatic maintenance, where the primary goals are to improve perfusion, preserve tissue function, and counter age-related physiologic decline.^30^^,^^31^ In contrast, CNV represents pathologic neovascularization driven by a chronic inflammatory microenvironment.^5^ Thus, NMN's effects may not be uniformly proangiogenic but instead may depend on tissue and disease context. In addition, previous studies have proposed that targeting SIRT1 may have therapeutic potential in CNV,^22^ supporting our findings that NMN likely modulates the CNV microenvironment primarily through regulation of NAD^+^-associated inflammatory pathways.

Accumulating evidence suggests that inflammatory signaling contributes to CNV progression, with NF-κB serving as a key mediator linking inflammatory cues to angiogenic responses.^23^^,^^32^^,^^33^ In our study, NMN downregulated the expression of NF-κB–regulated inflammatory mediators, including *Il1b*, *Tnfa*, *Il6*, *Ccl2*, and *Icam1*, in vivo and in vitro. These effects were associated with reduced NF-κB p65 phosphorylation and nuclear translocation, without altering total NF-κB protein levels. In addition, NMN treatment suppressed the activation of STAT3 and the expression of the stress-responsive protein HSP27. Collectively, NF-κB, STAT3, and HSP27 signaling have been implicated in inflammatory amplification and pathologic angiogenesis.^34^^–^^36^ Consistent with this anti-inflammatory profile, previous studies using LPS-induced inflammatory cell models have also supported the anti-inflammatory effects of NMN in macrophages and photoreceptor cells.^37^^,^^38^ Together, these findings demonstrate that NMN attenuates CNV development primarily by modulating the activation of inflammatory states, which may contribute to downstream alterations in cellular and tissue-level responses in CNV.

Importantly, suppression of inflammatory signaling was accompanied by marked cellular changes within the CNV microenvironment.^11^ NMN significantly reduced accumulation of myeloid cells within CNV lesions. Activated myeloid cells have been implicated in CNV persistence through enhanced cytokine production, sustained angiogenic signaling, and profibrotic remodeling.^7^^,^^10^ Together, our results indicate that NMN reduces intracellular inflammatory signaling and is accompanied by changes in the inflammatory cellular milieu within the CNV microenvironment.

Subretinal fibrosis is a major contributor to irreversible vision loss in nAMD.^3^ However, the laser-induced CNV model primarily represents an acute wound-healing response that undergoes spontaneous remodeling. Therefore, reduced collagen I deposition in vivo should be interpreted with caution, as it may partly reflect differences in lesion size resulting from reduced angiogenesis.^39^ To address this limitation, we employed a delayed treatment protocol in which NMN administration was initiated after the peak of CNV development, thereby minimizing the influence of early neovascular lesion formation on subsequent remodeling. Notably, delayed NMN treatment still significantly reduced lesion-associated collagen I deposition, suggesting that NMN may influence extracellular matrix remodeling during the later phase of lesion resolution. This interpretation is further supported by our in vitro findings, in which NMN suppressed TGF-β–induced profibrotic responses in THP-1–derived macrophages, including reduced α-SMA and collagen I expression, together with decreased expression of fibrosis-associated genes. Because macrophages exhibit substantial phenotypic plasticity and contribute to angiogenesis and tissue remodeling through inflammatory signaling and interactions with extracellular matrix components,^40^^,^^41^ these findings raise the possibility that NMN modulates macrophage-associated profibrotic responses in addition to its indirect effects through reducing CNV lesion development. Nevertheless, further studies using lineage-tracing approaches and temporal profiling will be required to define the spatiotemporal dynamics of macrophage phenotypic transitions during lesion remodeling.

From a translational perspective, NMN differs fundamentally from current anti-VEGF therapies. Whereas anti-VEGF agents selectively neutralize a single angiogenic driver,^42^ NMN appears to act more broadly by modulating upstream inflammatory and metabolic pathways that shape the CNV microenvironment. Notably, the efficacy of NMN in the delayed-treatment protocol suggests that it may remain effective even after angiogenic activity has peaked, a stage at which fibrosis and tissue remodeling increasingly dominate disease progression. These properties position NMN not as a replacement for anti-VEGF therapy but as a potential complementary strategy to improve long-term outcomes by modulating inflammatory activation and fibrosis-associated remodeling processes that are not adequately addressed by VEGF blockade alone.

Several limitations should be acknowledged. The laser-induced CNV model is an acute injury paradigm with spontaneous lesion resolution and does not fully recapitulate the chronic, aging-associated pathology of human nAMD. In addition, although NMN modulates NAD^+^-dependent signaling and inflammatory activation,^13^^,^^43^ the specific cell types and downstream mediators that drive its in vivo effects remain to be determined. Future studies using aging models, cell type–specific approaches, and combination regimens with anti-VEGF agents will be essential to more precisely define NMN's therapeutic potential.

## Conclusions

Our findings suggest that NMN attenuates inflammatory signaling, reduces inflammatory cell accumulation within CNV lesions, and limits lesion-associated extracellular matrix remodeling. By modulating upstream inflammatory and metabolic pathways rather than a single angiogenic effector, NMN may represent a promising adjunctive therapeutic strategy to complement anti-VEGF therapy and improve long-term outcomes in nAMD.

## Supplementary Material

Supplement 1
