# Supplementary material for: Nicotinamide Mononucleotide Attenuates Inflammatory Activation, Choroidal Neovascularization, and Lesion-Associated Remodeling
Source: Invest Ophthalmol Vis Sci. 2026 Aug 25;67(10):59. doi: 10.1167/iovs.67.10.59 (PMC13543604; doi:10.1167/iovs.67.10.59)
Supplement: Supplementary file 1 [file iovs-67-10-59_s001.pdf]

Supplements

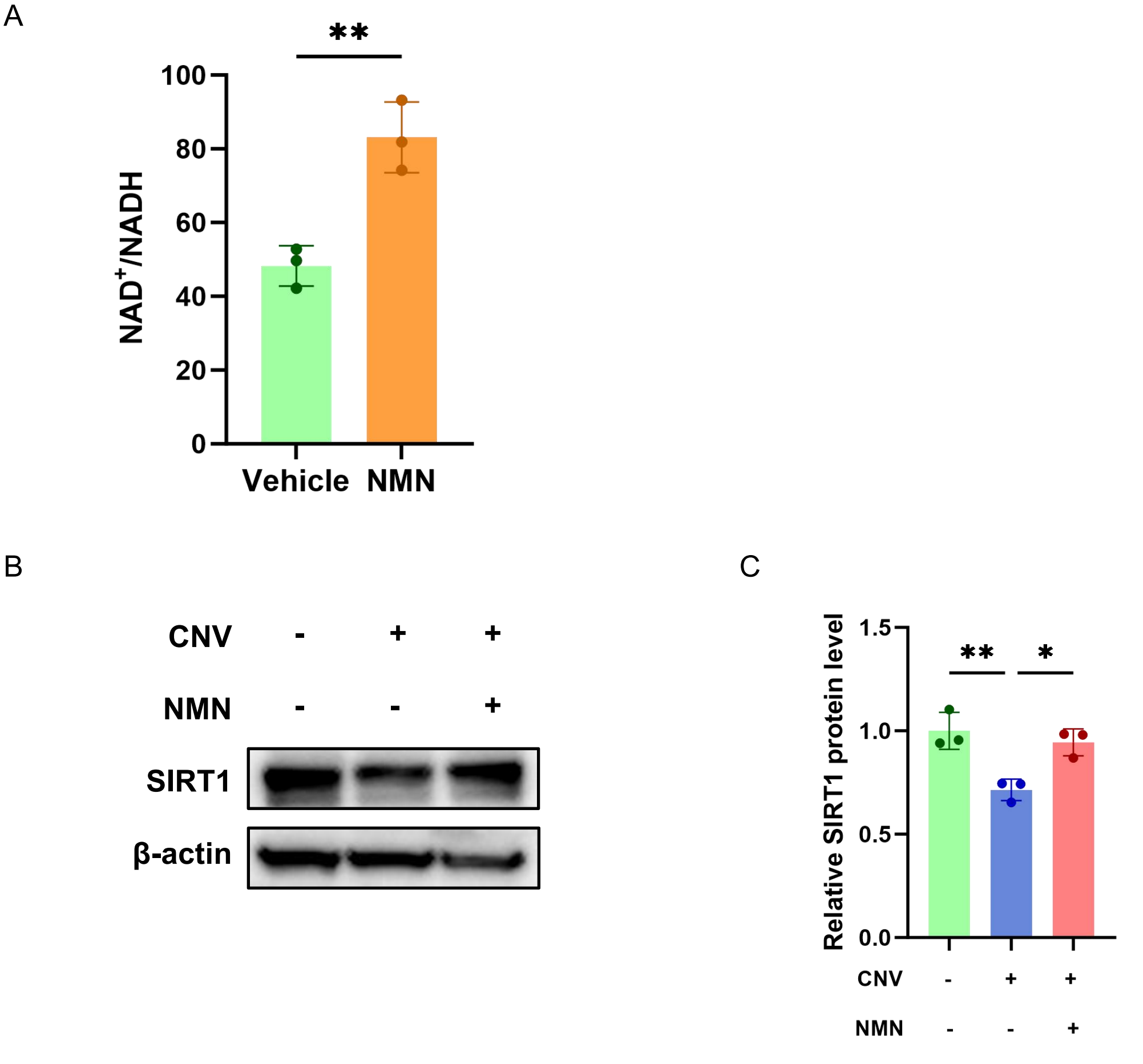

Supplementary Fig. S1. NMN elevates the retinal NAD$^+$ level and restores SIRT1 expression.

(A) Quantification of the retinal NAD$^+$/NADH ratio. Retinas were pooled (6 retinas per sample; n = 3).

(B, C) Western blot analysis of neural retina. Representative immunoblots are shown in (B). Densitometric quantification of SIRT1 is shown in (C) (n = 3). Proteins were normalized to β-actin.

Data are presented as mean ± SD. Student's *t*-test in (A); One-way ANOVA in (C). *$P < 0.05$, **$P < 0.01$.

(Fig.4A)

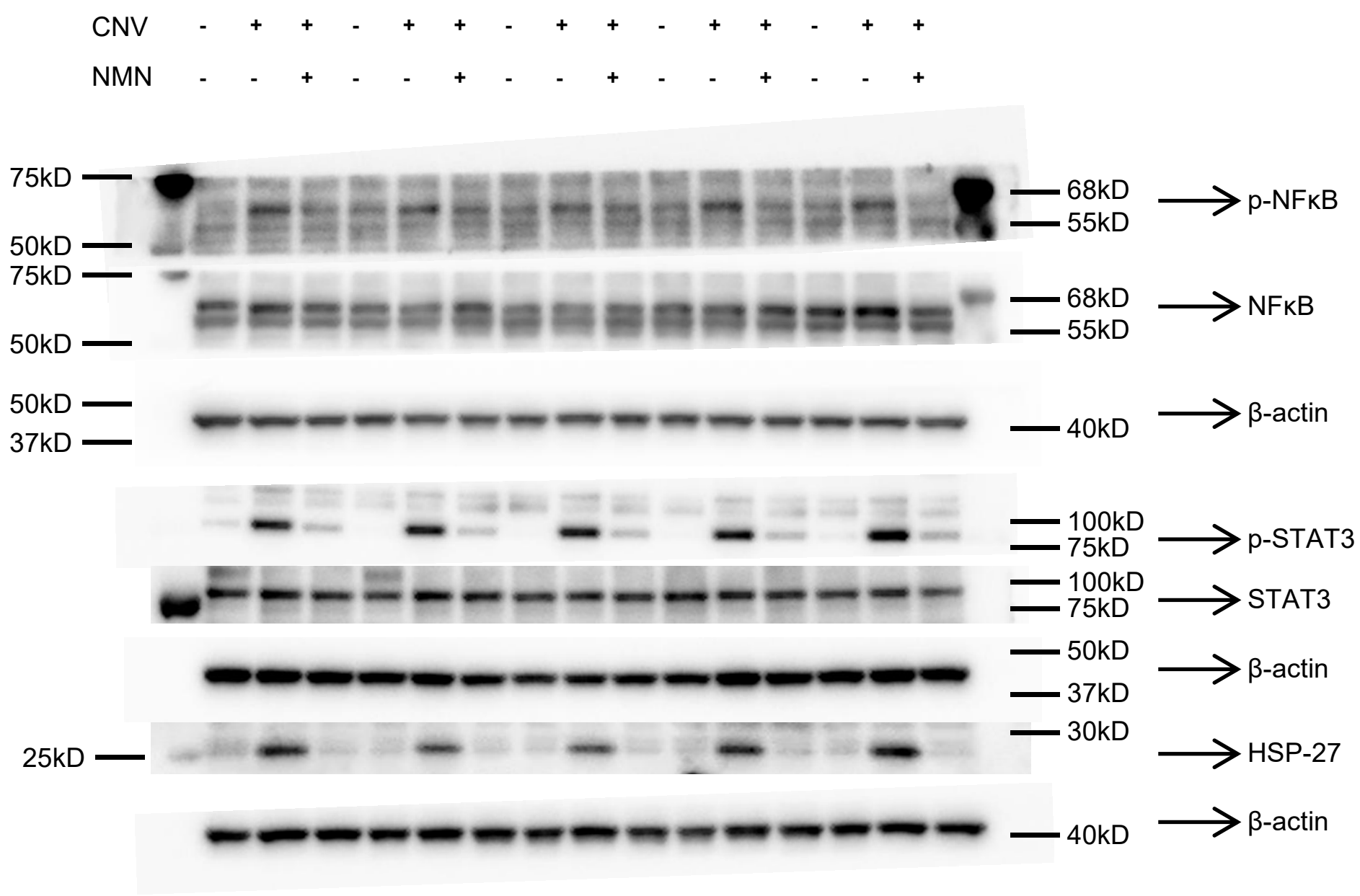

(Fig.6E)

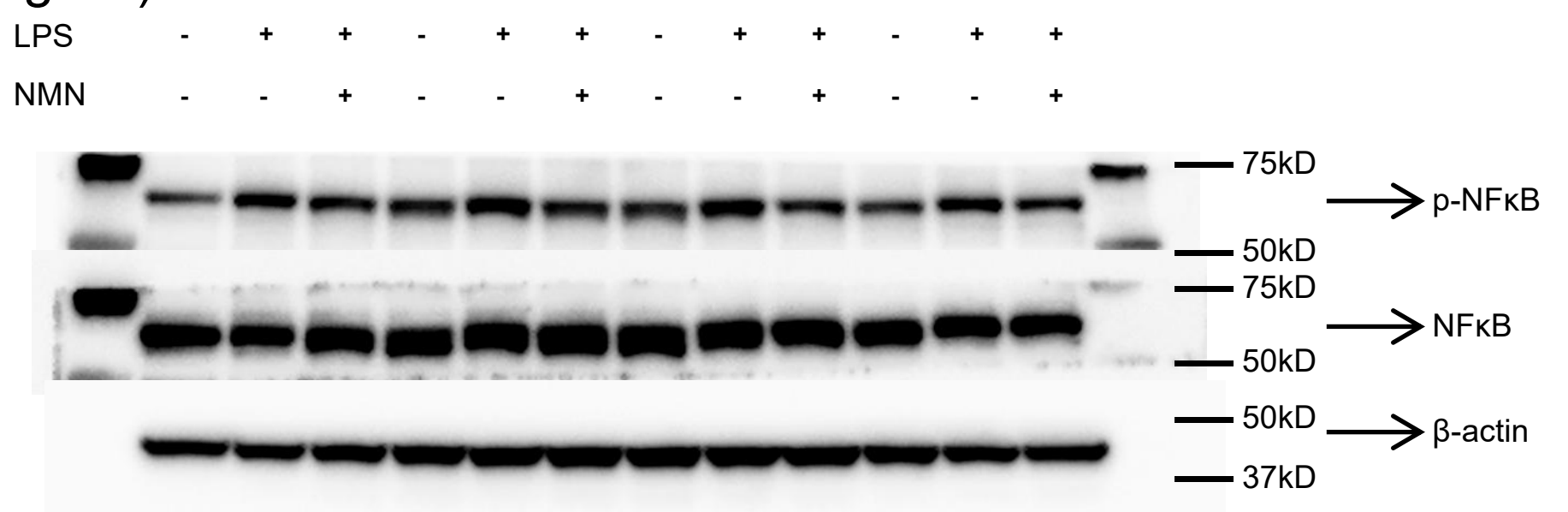

(Fig.S1)

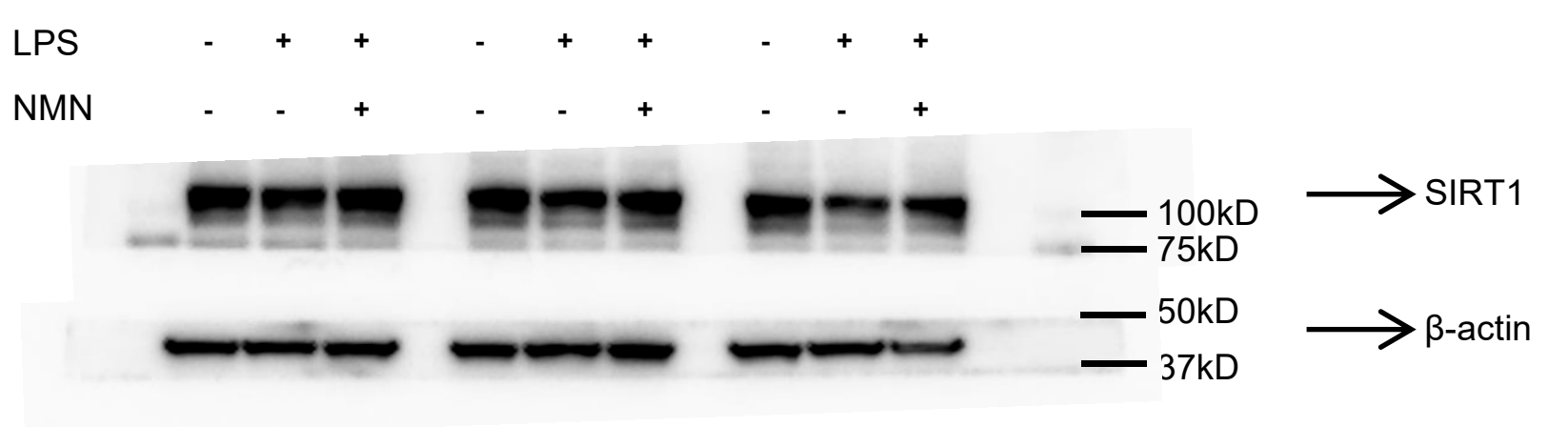

Supplementary Fig. S2. Raw images of western blot for all proteins analyzed.
